# Prioritization of ICU beds with renal replacement therapy support by court order and mortality in a Brazilian metropolitan area

**DOI:** 10.1038/s41598-022-07429-4

**Published:** 2022-03-03

**Authors:** Ana Cristina dos Santos, Simone Luzia Fidelis de Oliveira, Virgílio Luiz Marques de Macedo, Paula Lauane Araujo, Francine Salapata Fraiberg, Nélliton Fernandes Bastos, Richard Lucas Alves, Carlos Darwin Gomes da Silveira, Sérgio Eduardo Soares Fernandes, Francisco de Assis Rocha Neves, Fábio Ferreira Amorim

**Affiliations:** 1 Graduation Program in Health Sciences, Universidade de Brasília (UnB), SMHN Quadra 03, Conjunto A, Bloco 1, Edifício FEPECS, Brasília, Federal District CEP: 70710-907 Brazil; 2 Nursing School, School of Health Sciences, Escola Superior de Ciências da Saúde (ESCS), Brasília, DF Brazil; 3 Regulation Center of Federal District, Secretaria de Saúde do Distrito Federal, Brasília, DF Brazil; 4 Graduation Program in Nursing, Universidade de Brasília (UnB), Brasília, DF Brazil; 5 Multiprofessional Residency Program in Primary Care, Fundação Oswaldo Cruz (FIOCRUZ), Brasília, DF Brazil; 6 Law School, Centro Universitário do Distrito Federal (UDF), Brasília, DF Brazil; 7 Medical School, School of Health Sciences, Escola Superior de Ciências da Saúde (ESCS), Brasília, Brasília, Federal District Brazil

**Keywords:** Health care, Medical research

## Abstract

The shortage of intensive care unit (ICU) resources, including equipment and supplies for renal replacement therapy (RRT), is a critical problem in several countries. This study aimed to assess hospital mortality and associated factors in patients treated in public hospitals of the Federal District, Brazil, who requested admission to ICU with renal replacement therapy support (ICU-RRT) in court. Retrospective cohort study that included 883 adult patients treated in public hospitals of the Federal District who requested ICU-RRT admission in court from January 2017 to December 2018. ICU-RRT was denied to 407 patients, which increased mortality (OR 3.33, 95% CI 2.39–4.56, *p* ≪ 0.01), especially in patients with priority level I/II (OR 1.02, 95% CI 1.01–1.04, *p* ≪ 0.01). Of the requests made in court, 450 were filed by patients with priority levels III/IV, and 44.7% of these were admitted to ICU-RRT. In admitted patients, priority level III priority level I/II was associated with a low mortality (OR 0.47, 95% CI 0.32–0.69, *p* < 0.01), and not. The admission of patients classified as priority levels III/IV to ICU-RRT considerably jeopardized the admission of patients with priority levels I/II to these settings. The results found open new avenues for organizing public policies and improving ICU-RRT triage.

## Introduction

The shortage of intensive care unit (ICU) resources, including equipment and supplies for renal replacement therapy (RRT), is a critical problem in several countries^1–8^ even before the COVID-19 pandemic, drawing attention to the limited capacity of resources in ICU settings worldwide^9–17^. The demand for ICU beds with RRT support (ICU-RRT) exceeding the available capacity is a crucial issue in Brazil, mainly in public hospitals, postponing ICU admission and the RRT initiation^6^ and affecting outcomes, such as hospital length of stay and mortality^18–23^.

As acute kidney injury (AKI) is among the most frequent organ failure conditions and a serious complication in patients critically ill from multiple and varied etiologies, being associated with poor outcomes, including a high mortality rate^9,24–27^, improving the health care delivery for patients with AKI is essential. Indeed, an international multicenter study performed in 97 ICUs worldwide showed that AKI occurred in over half of ICU patients and that 13.5% of critically ill patients needed RRT during the first week of ICU stay^25^. Since the RRT benefits in reducing mortality and life-threatening complications in patients with severe AKI are unequivocal, the shortage of ICU-RRT beds is a critical problem^18,28^.

In a health system with limited resources to meet the increased demand for ICU and RRT, ICU admission regulation centers were created in each Brazilian state to control and optimize the flow of ICU admissions in public hospitals. These centers define and control the waiting list for ICU admission according to a priority scheme based on the disease severity, on whether another support therapy is required, such RRT, on the possible benefit from ICU admission, and on the time of ICU admission request^7,8,29^.

The Brazilian Federal Constitution guarantees access to a public and universal health system to all Brazilians^30,31^. Since there is an excessive demand for ICU-RRT and a delay in admission, many patients wait days for an ICU-RRT bed and, sometimes, never get the chance to be admitted; while others request an ICU-RRT bed in court even when they have lower priority on the waiting list of regulation centers^30,31^.

In this circumstance, these judicial litigations may lead to the admission of patients classified as lower priority levels regardless of their waiting list position, affecting the ICU-RRT admission of patients who would benefit the most from this treatment. Besides improving the triage system, it is essential to analyze if the ICU-RRT admission of low-priority patients makes a difference in their outcome when compared to that of non-ICU-RRT admitted patients. Thus, the primary purpose of this study was to assess hospital mortality in patients who requested ICU-RRT admission in court due to the scarcity of ICU beds in the Brazilian public health system. As a secondary purpose, it aimed to assess the ICU admission effect on hospital mortality according to the patients' ICU-RRT priority level, as well as factors associated with denied ICU-RRT admission and hospital mortality in all patients, ICU-RRT admitted patients, and non-ICU-RRT admitted patients.

## Materials and methods

This is a retrospective cohort study including all consecutive patients older than 18 years treated in the public hospitals or emergency care units of the Federal District, Brazil, who needed to request ICU-RRT admission in court from January 2017 to December 2018.

The Federal District is a metropolitan area with 2,469,489 inhabitants. It is serviced by a local public health system that has 15 hospitals, 12 of them with ICU settings (13 general ICUs, one trauma ICU, and one coronary ICU) and six emergency care units. The allocation of ICU beds in any setting of the Federal District public health system is coordinated by the Regulation Center of the state of Goiás. In this process, a physician defines a priority level for each patient based on their disease severity and on the possible patient benefit from ICU admission. As the demand for ICU-RRT beds is higher than the number of beds available in the public health system, patients are placed on a waiting list according to their priority level and to the time when the ICU-RRT bed was requested. Due to the delay in admission, some patients demand it in court regardless of their waiting list position, which can lead to the admission of patients in disregard of the priorities defined by the Regulation Center.

**Figure 1:**
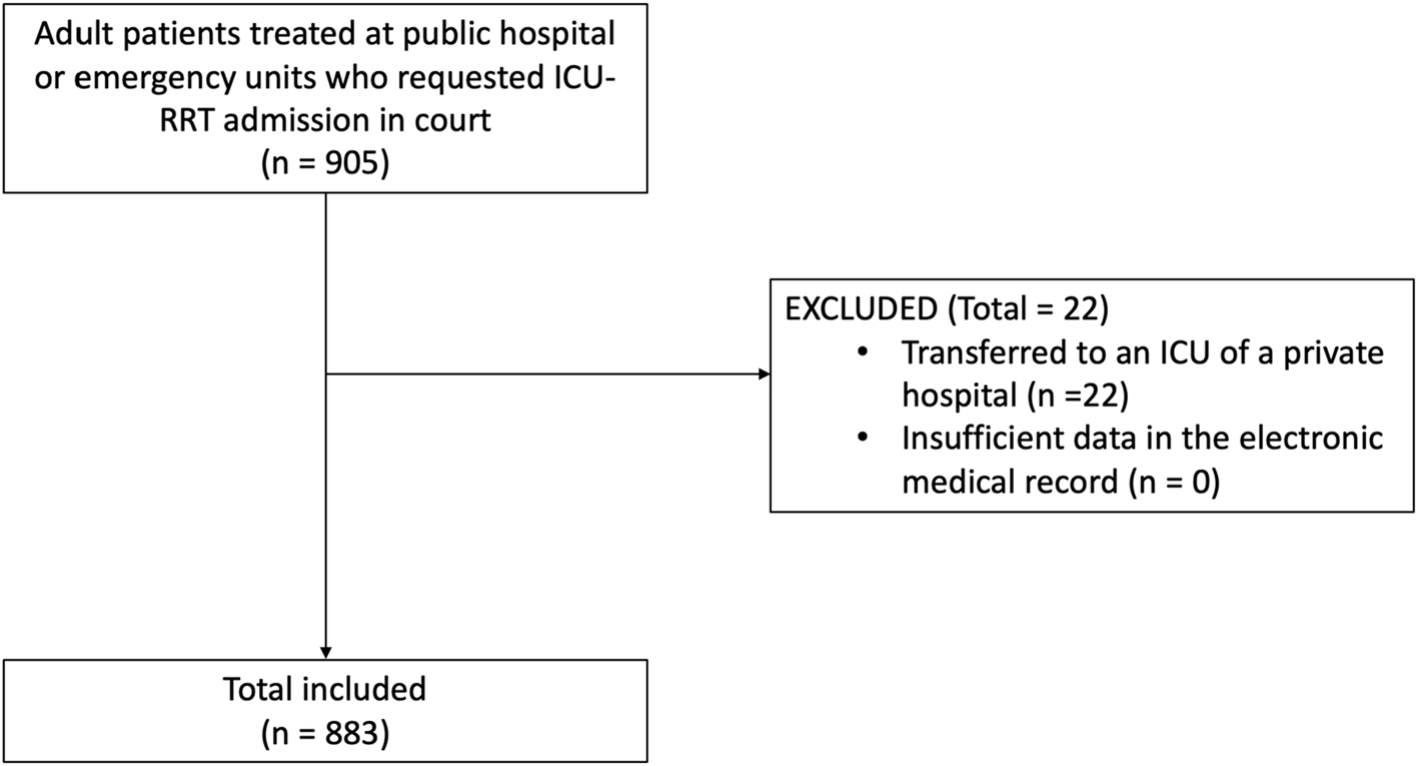
Flowchart of the study patient selection process.

The inclusion criteria to assess hospital mortality in patients who requested ICU-RRT admission in court were patients aged 18 years or older treated in public hospitals or emergency care units in the Federal District who requested ICU-RRT admission in court during the study period. The exclusion criteria were patients transferred to private hospital ICUs or insufficiency of data in the electronic medical record of the Federal District public health system. In this respect, 905 adult patients treated in public hospitals or emergency units requested ICU-RRT admission in court due to scarcity of ICU-RRT beds. Twenty-two patients were transferred to private hospital ICUs (2.5%). No patient was excluded due to insufficiency of data in the electronic medical record. Thus, 883 patients were included in the study, Fig. 1. The sample distribution according to the public health service in which patients were treated at the time the ICU-RRT admission was requested in court is shown in Supplementary Table S1.

The variables collected were age, gender, primary reason for hospital admission, priority level classification at the time of ICU-RRT request according to the Regulation Center that services the Federal District, whether ICU-RRT admission occurred or not, and outcome during the hospital stay (survivors versus non-survivors). The outcomes evaluated were denial of ICU-RRT admission and hospital mortality in all patients, ICU-RRT admitted patients, and non-ICU-RRT admitted patients. Patients were not followed up after hospital discharge.

The priority level classification applied by the Regulation Center is described below^32^:

- Priority level I—critically ill patients who require intensive care, should benefit from immediate intensive care/life support interventions, and do not have limitations of care;
- Priority level II—critically ill patients without hemodynamic instability who require intensive monitoring and care due to the risk of rapid decompensation. They may potentially need immediate intervention and do not have limitations of care;
- Priority level III—critically ill patients who may require intensive care but have a reduced chance of survival from the underlying disease, the nature of their acute illness, or comorbidities. Intensive treatment may alleviate the critical condition; however, there may be limits on therapeutic efforts, such as intubation or cardiopulmonary resuscitation;
- Priority IV—patients with terminal illnesses or who should benefit from palliative care rather than inappropriately aggressive or heroic interventions and patients who are in good conditions to benefit from ICU admission and are at low risk of needing an intervention that should be performed in an ICU setting.

The criteria for priority level classification according to the Regulation Center that services the Federal District is detailed in Supplementary Table S2.

Categorical variables (priority levels, gender, primary reason for hospital admission, denial of ICU-RRT admission, and hospital mortality) are expressed as number and percentage (%). For age (quantitative variable), data are expressed as mean ± standard deviation (SD) and the median and interquartile range 25–75% (IQ 25–75). There were no outliers or missing data. Additionally, there was no need to use censoring strategies in this study.

Considering the outcomes to be analyzed (denial of ICU-RRT admission and hospital mortality in all patients, ICU-RRT admitted patients, and non-ICU-RRT admitted patients), patients were grouped according to the independent variable studied. Contingency tables were used for categorical variables, and Pearson's Chi-square test (χ^2^) or Fisher's exact test were used as appropriate. For being a continuous variable, Mann–Whitney tests were used to compare age among groups because the Kolmogorov–Smirnov test with Lilliefors correction showed a non-parametric distribution (*p* < 0.01). Post-hoc analysis was performed using adjusted residuals with Bonferroni correction, as indicated.

To evaluate independent factors associated with denial of ICU-RRT admission and hospital mortality in all patients, ICU-RRT admitted patients, and non-ICU-RRT admitted patients, a binary logistic regression analysis with enter method was performed including outcome-associated non-collinear variables, with a *p*-value < 0.05 in the univariate analysis, and confounding factors according to previous knowledge, with a *p*-value < 0.20 in the univariate analysis. Non-collinearity was accepted when the tolerance was higher than 0.10 and the variance inflation factor (VIF) was lower than 10.0. Factors included in the logit regression for denial of ICU-RRT admission were age, priority level I/II classification, respiratory conditions as the primary reason for hospital admission, and trauma as the primary reason for hospital admission. Factors included in the logit regression for hospital mortality in all patients were age, priority level I/II classification, denial of ICU-RRT admission, renal conditions as the primary reason for hospital admission, cardiovascular conditions as the primary reason for hospital admission, and trauma as the primary reason for hospital admission. Factors included in the logit regression for mortality in ICU-RRT admitted patients were age, priority level I/II classification, renal conditions as the primary reason for hospital admission, and digestive conditions as the primary reason for hospital admission. Factors included in the logit regression for mortality in non-ICU-RRT admitted patients were age, priority level I/II classification, renal conditions as the primary reason for hospital admission, and digestive conditions as the primary reason for hospital admission.

To assess the ICU admission effect on hospital mortality according to the priority level classification, a propensity score matching for ICU-RRT admission was performed applying a logit regression model adjusted for factors independently associated with denial of ICU-RRT admission or hospital mortality in all patients (age, priority level classification, and renal conditions as the primary reason for hospital admission). The EZR software version 1.54 (Saitama Medical Center, Jichi Medical University, Japan) with a 1:1 pair-matching ratio without replacement on the logit of the propensity score was employed in this analysis using a caliper of 0.2 width. Supplementary Table S3 shows the sample before and after matching. The odds ratio (OR) and 95% confidence interval (95% CI) of the ICU admission effect on hospital mortality according to the priority level classification was calculated in the sample after matching, being shown as a forest plot. The estimates for the ICU admission effect on hospital mortality found through propensity score analysis showed an OR of 0.29 (95% CI 0.21–0.40).

Statistical analyses were performed using IBM Statistical Package for Social Sciences 20.0 for Mac (SPSS 20.0 Mac, SPSS Inc., Chicago, USA), statistical software R version 4.0.5 (R Foundation for Statistical Computing), and EZR software version 1.54 (Saitama Medical Center, Jichi Medical University, Japan). The level of statistical significance was defined as a two-sided *p*-value ≤ 0.05.

The Ethics Committee of the Education and Research Foundation of Health Sciences (FEPECS), Brasília, Federal District, Brazil, approved the study under opinion number 3.575.349. The study was conducted following the Declaration of Helsinki. Since this is a retrospective cohort study, having no specific intervention, but only using anonymized medical record data and other institutional clinical information that generated results in an aggregate manner, not allowing the identification of participants, the written consent was not necessary according to the Resolution of the Brazilian National Research Ethics Council.

## Results

Of the 883 patients included in the study, 255 were classified as priority level I (28.9%); 178, as priority level II (20.2%); 394, as priority level III (44.6%); and 56, as priority level IV (6.3%). The mean age was 65.9 ± 15.6 years, 43.8% of patients were female (387/883), and 46.1% of patients were not admitted to ICU-RRT (407/883), even after court order. The main primary reasons for hospital admission were related to cardiovascular (392/883, 44.4%) and respiratory (228/883, 25.8%) conditions, Supplementary Table S4.

### Denial of ICU-RRT admission

Of the 407 patients with denied ICU-RRT admission, 39.6% were classified as priority level I (101/255); 38.8%, as priority level II (69/178); 49.5%, as priority level III (195/394); and 75.0%, as priority level IV (42/56). There was a significant difference in denial of ICU-RRT admission considering the priority level classification, *p* < 0.01, Supplementary Table S5.

Table 1 shows the univariate analysis of variables associated with denial of ICU-RRT admission. Patients with denied admission are older than those admitted to ICU-RRT (67.7 ± 15.8 years versus 64.4 ± 15.2 years, *p* ≤ 0.01). Denial of ICU-RRT admission was lower in patients classified as priority levels I/II than in patients classified as priority levels III/IV (41.8% versus 55.3%, *p* < 0.01). There was no significant difference in denial of ICU-RRT admission considering the primary reason for hospital admission and gender.

**Table 1:** Univariate analysis of variables associated with denial of ICU-RRT admission in patients who requested ICU-RRT admission in court (n = 883).

Variables	Denial of ICU-RRT admission (n = 407)	With ICU-RRT admission (n = 476)	OR (95% CI)	*p-*value
**Age, years**				
Mean (SD)	67.7 (15.2)	64.4 (15.2)	1.01 (1.01–1.02)	< 0.01
Median (IQ 25–75%)	66.0 (55.0–76.0)	70.0 (59.0–79.0)		
Priority levels I/II, n (%)	170 (41.8)	263 (55.3)	0.58 (0.44–0.76)	< 0.01
Female gender, n (%)	189 (46.4)	198 (41.6)	1.22 (0.93–1.59)	0.15
**Primary reason for hospital admission, n (%)**				
Cardiovascular	189 (46.4)	203 (42.6)	1.17 (0.89–1.52)	0.26
Respiratory	96 (23.6)	132 (27.7)	0.80 (0.59–1.09)	0.16
Renal	44 (10.8)	49 (10.3)	1.06 (0.69–1.62)	0.80
Neurological	31 (7.6)	37 (7.8)	0.98 (0.60–1.61)	0.93
Digestive	34 (8.4)	29 (6.1)	1.40 (0.84–2.35)	0.91
Trauma	10 (2.5)	20 (4.2)	0.57 (0.27–1.24)	0.15
Others	3 (0.7)	6 (1.3)	0.58 (0.14–2.34)	0.44

*ICU-RRT* Intensive care unit with renal replacement therapy support, *OR* Odds ratio, *95% CI* 95% confidence interval, *SD* standard deviation, *IQ 25–75% *interquartile range 25–75%.

Table 2 shows the multivariate analysis of variables associated with denied ICU-RRT admission. Age (OR 1.01, 95% CI 1.01–1.02, *p* ≤ 0.01) was independently associated with a high rate of denial of ICU-RRT, while priority levels I/II (OR 0.58, 95% CI 0.44–0.76, *p* < 0.01) were independently associated with a low rate of denial of ICU-RRT.

**Table 2:** Multivariate analysis of variables associated with denial of ICU-RRT admission in patients who requested ICU-RRT admission in court (n = 883).

Variables	Collinearity statistics		Binary logistic regression	
	Tolerance	VIF	OR (95% CI)	*p-*value
Age (per year)	1.00	1.00	1.01 (1.01–1.02)	< 0.01
Priority levels I/II	0.99	1.02	0.58 (0.44–0.76)	< 0.01
Respiratory primary reason for hospital admission	0.98	1.02	0.74 (0.54–1.01)	0.06
Trauma primary reason for hospital admission	0.98	1.02	0.56 (0.26–1.24)	0.15

*ICU-RRT* Intensive care unit with renal replacement therapy support, *VIF* variance inflation factor, *OR* Odds ratio, *95% CI* 95% confidence interval.

### Hospital mortality in all patients

Hospital mortality considering all patients was 69.3% (612/883): 71.0% of patients with priority level I (181/255); 56.7% of patients with priority level II (101/178); 78.7% of patients with priority level III (310/394); and 35.7% of patients with priority level IV (20/56). There was a significant difference in hospital mortality considering the priority level classification, *p* < 0.01, Supplementary Table S6.

Table 3 shows the univariate analysis of variables associated with hospital mortality. Patients with denied ICU-RRT admission showed a higher hospital mortality than patients admitted to ICU-RRT (55.1% versus 25.8%, *p* < 0.01). Non-survivors were older than survivors (67.5 ± 14.9 years versus 62.1 ± 16.9 years, *p* < 0.01). Hospital mortality was lower in patients classified as priority levels I/II than in patients classified as priority levels III/IV (46.1% versus 55.7%, *p* < 0.01). Among the primary reasons for hospital admission, hospital mortality was lower in patients with renal conditions (8.8% versus 14.4%, *p* = 0.01) and trauma conditions (2.6% versus 5.2%, *p* = 0.01).

**Table 3:** Univariate analysis of variables associated with hospital mortality in patients who requested ICU-RRT admission in court (n = 883).

Variables	Non-survivors (n = 612)	Survivors (n = 271)	OR (95% IC)	*p-*value
**Age, years**				
Mean (SD)	67.5 (14.9)	62.1 (16.9)	1.01 (1.01–1.02)	< 0.01
Median (IQ 25–75%)	64.0 (51.0–73.0)	70.0 (58.2–79.0)		
Priority levels I/II, n (%)	282 (46.1)	151 (55.7)	0.58 (0.44–0.76)	< 0.01
Denial of ICU-RRT admission, n (%)	337 (55.1)	70 (25.8)	3.52 (2.57–4.82)	< 0.01
Female gender, n (%)	280 (45.8)	107 (39.5)	1.29 (0.97–1.73)	0.08
**Primary reason for hospital admission, n (%)**				
Cardiovascular	282 (46.1)	110 (40.6)	1.25 (0.94–1.67)	0.13
Respiratory	160 (26.1)	68 (25.1)	1.06 (0.76–1.47)	0.74
Renal	54 (8.8)	39 (14.4)	0.58 (0.37–0.89)	0.01
Neurological	47 (7.7)	21 (7.7)	0.99 (0.58–1.69)	0.97
Digestive	48 (7.8)	15 (5.5)	1.45 (0.80–2.64)	0.22
Trauma	16 (2.6)	14 (5.2)	0.49 (0.24–1.02)	0.05
Others	5 (0.8)	4 (1.5)	0.55 (0.15–2.06)	0.37

*ICU-RRT* Intensive care unit with renal replacement therapy support, *OR* Odds ratio, *95% CI* 95% confidence interval, *SD* standard deviation, *IQ 25–75% *interquartile range 25–75%.

Table 4 shows the multivariate analysis of variables associated with hospital mortality. Age (OR 1.02, 95% CI 1.01–1.03, *p* < 0.01) and denial of ICU-RRT admission (OR 3.33, 95% CI 2.39–4.56, *p* < 0.01) were independently associated with a high hospital mortality. Renal conditions as the primary reason for hospital admission (OR 0.56, 95% CI 0.34–0.91, *p* = 0.02) was independently associated with a low hospital mortality.

**Table 4:** Multivariate analysis of variables associated with hospital mortality in patients who requested ICU-RRT admission in court (n = 883).

Variables	Collinearity statistics		Binary logistic regression	
	Tolerance	VIF	OR (95% CI)	*p-*value
Age (per year)	0.98	1.01	1.02 (1.01–1.03)	< 0.01
Priority levels I/II	0.97	1.03	0.81 (0.60–1.10)	0.18
Denial of ICU-RRT admission	0.97	1.03	3.30 (2.39–4.56)	< 0.01
Renal primary reason for hospital admission	0.89	1.12	0.56 (0.34–0.91)	0.02
Cardiovascular primary reason for hospital admission	0.86	1.16	1.05 (0.76–1.46)	0.76
Trauma primary reason for hospital admission	0.95	1.05	0.51 (0.23–1.12)	0.09

*ICU-RRT* Intensive care unit with renal replacement therapy support, *VEF* variance inflation factor, *OR* Odds ratio, *95% CI* 95% confidence interval.

### Hospital mortality in patients with ICU-RRT admission

Of the patients with ICU-RRT admission, 57.8% died (275/476): 53.2% of patients with priority level I (82/154); 45.0% of patients with priority level II (49/109); 69.3% of patients with priority level III (138/199); and 42.9% of patients with priority level IV (6/14). There was a significant difference in hospital mortality considering the priority level classification, *p* < 0.01, Supplementary Table S6.

In the univariate analysis of factors associated with hospital mortality in patients with ICU-RRT admission, non-survivors were older than survivors (66.0 ± 14.8 years versus 62.1 ± 15.6 years, *p* < 0.01). Hospital mortality was lower in patients classified as priority levels I/II than in patients classified as priority levels III/IV (47.6% versus 65.7%, *p* < 0.01). Regarding the primary reason for hospital admission, hospital mortality was lower in patients with renal conditions (7.3% versus 13.4%, *p* = 0.05) and higher in patients with digestive conditions (8.0% versus 5.5%, *p* = 0.04) (Table 5).

**Table 5:** Univariate analysis of factors associated with hospital mortality in patients with ICU-RRT admission (n = 476) and patients with denied ICU-RRT admission (n = 407).

Variables	Patients with ICU-RRT admission				Patients with denied ICU-RRT admission			
	Non-survivors (n = 275)	Survivors (n = 201)	OR (95% IC)	*p* value	Non-survivors (n = 337)	Survivors (n = 70)	OR (95% IC)	*p-*value
**Age, years**								
Mean (SD)	66.0 (14.8)	62.1 (15.6)	1.01 (1.01–1.02)	< 0.01	68.8 (14.9)	62.1 (18.8)	1.02 (1.01–1.04)	< 0.01
Median (IQ 25–75%)	68.0 (56.0–77.0)	64.0 (52.0–73.0)			71.0 (60.5–80.0)	65.0 (50.8–75.2)		
Priority levels I/II	131 (47.6)	132 (65.7)	0.48 (0.33–0.69)	< 0.01	151 (44.8)	19 (27.1)	2.18 (1.24–3.85)	0.01
Female gender, n (%)	117 (42.5)	81 (40.3)	1.22 (0.93–1.59)	0.62	163 (48.4)	26 (37.1)	1.58 (0.93–2.69)	0.09
**Primary reason for hospital admission, n (%)**								
Cardiovascular	121 (44.0)	82 (40.8)	1.17 (0.8–1.52)	0.48	161 (47.8)	28 (40.0)	1.37 (0.81–2.32)	0.24
Respiratory	79 (28.7)	53 (26.4)	0.80 (0.59–1.09)	0.57	81 (24.0)	15 (21.4)	1.16 (0.62–2.16)	0.64
Renal	20 (7.3)	27 (13.4)	1.06 (0.69–1.62)	0.05	32 (9.5)	12 (17.1)	0.51 (0.25–1.04)	0.06
Neurological	9 (3.3)	17 (8.5)	1.40 (0.84–2.35)	0.63	27 (8.0)	4 (5.7)	1.44 (0.49–4.24)	0.51
Digestive	22 (8.0)	11 (5.5)	1.40 (0.84–2.35)	0.04	7 (2.1)	3 (4.3)	0.65 (0.28–1.50)	0.31
Trauma	22 (8.0)	7 (3.5)	0.57 (0.27–1.24)	0.24	26 (7.7)	8 (11.4)	0.47 (0.12–1.88)	0.28
Others	2 (0.7)	4 (2.0)	0.58 (0.14–2.34)	0.22	3 (0.01)	0 (0.0)		0.57

*ICU-RRT* Intensive care unit with renal replacement therapy support, *OR* Odds ratio, *95% CI* 95% confidence interval, *SD* standard deviation, *IQ 25–75% *interquartile range 25–75%.

In the multivariate analysis of factors associated with hospital mortality in patients with ICU-RRT admission, age (OR 1.02, 95% CI 1.01–1.03, *p* = 0.02) and digestive conditions as the primary reason for hospital admission (OR 2.53, 95% CI 1.04–6.15, *p* = 0.04) were independently associated with a high hospital mortality. Priority levels I/II (OR 0.47, 95% CI 0.32–0.69, *p* < 0.01) were independently associated with a low hospital mortality (Table 6).

**Table 6:** Multivariate analysis of factors associated with hospital mortality in patients with ICU-RRT admission (n = 476) and patients with denied ICU-RRT admission (n = 407).

Variables	Collinearity statistics		Binary logistic regression	
	Tolerance	VEF	OR (95% CI)	*p-*value
**Hospital mortality in patients with ICU-RRT admission**				
Age (per year)	0.99	1.01	1.02 (1.01–1.03)	0.02
Priority levels I/II	0.99	1.01	0.47 (0.32–0.69)	< 0.01
Renal primary reason for hospital admission	0.98	1.02	0.06 (0.35–1.19)	0.16
Digestive primary reason for hospital admission	0.99	1.01	2.53 (1.04–6.15)	0.04
**Hospital mortality in patients with denied ICU-RRT admission**				
Age (per year)	1.00	1.00	1.03 (1.01–1.04)	< 0.01
Priority levels I/II	1.00	1.00	2.22 (1.25–3.96)	< 0.01
Renal primary reason for hospital admission	1.00	1.00	0.49 (0.23–1.03)	0.06

*ICU-RRT* Intensive care unit with renal replacement therapy support, *VEF* variance inflation factor, *OR* Odds ratio, *95% CI* 95% confidence interval.

### Hospital mortality in patients with denied ICU-RRT admission

Of the patients with denied ICU-RRT admission, 82.8% died (337/407): 98.0% of patients with priority level I (99/101); 75.4% of patients with priority level II (52/69); 88.2% of patients with priority level III (172/195); and 33.3% of patients with priority level IV (14/42). There was a significant difference in hospital mortality considering the priority level classification, *p* < 0.01, Supplementary Table S6.

In the univariate analysis of factors associated with hospital mortality in patients with denied ICU-RRT admission, non-survivors were older than survivors (66.8 ± 14.9 years versus 62.1 ± 18.8 years, *p* < 0.01). Hospital mortality was higher in patients classified as priority levels I/II than in patients classified as priority levels III/IV (44.8% versus 27.1%, *p* < 0.01). There was no significant difference regarding the primary reasons for hospital admission or gender in hospital mortality.

In the multivariate analysis of factors associated with hospital mortality in patients with denied ICU-RRT admission, age (OR 1.02, 95% CI 1.01–1.04, *p* < 0.01) and priority levels I/II (OR 2.18, 95% CI 1.24–3.85, *p* = 0.01) were independently associated with a high mortality (Table 6).

### ICU admission effect on hospital mortality according to the priority level classification

**Figure 2:**
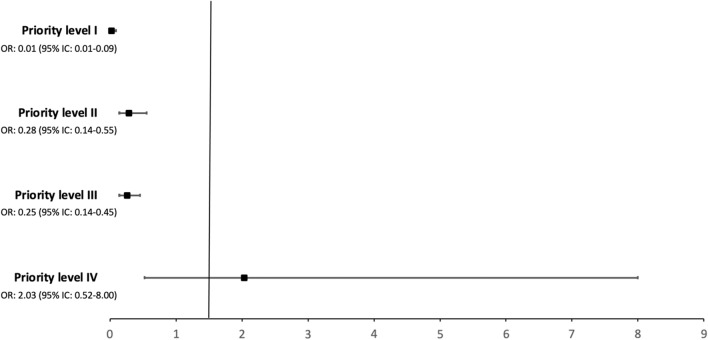
ICU admission effect on hospital mortality according to the sample priority level classification after propensity score-matching analysis for ICU-RRT admission adjusted to age, priority level classification and renal primary reason for hospital admission.

Figure 2 shows the analysis of the ICU admission effect on hospital mortality according to the sample priority level classification after the propensity score matching analysis for ICU-RRT admission adjusted for age, priority level classification, and renal conditions as the primary reason for hospital admission. ICU admission was associated with a lower hospital mortality among patients classified as priority level I than among patients classified as other priority levels (*p* < 0.01). ICU admission was associated with a significantly low hospital mortality among patients classified as priority level I (OR 0.01, 95% CI 0.01–0.09, *p* < 0.01), priority level II (OR 0.28, 95% CI 0.14–0.55, *p* < 0.01), and priority level III (OR 0.25, 95% CI 0.14–0.45, *p* < 0.01), but not as priority level IV (OR 2.03, 95% CI 0.52–8.00, *p* = 0.31).

## Discussion

In this study, 40.9% of patients who requested ICU-RRT admission in court did not succeed in their request. This finding shows that the shortage of ICU-RRT beds is a critical problem in Brazil since before the COVID-19 pandemic reached the country (February 26, 2020)^33^. Patients without ICU-RRT admission showed a significantly higher mortality than ICU-RRT admitted patients. In particular, this fact is crucial in patients without ICU-RRT admission classified as priority level I, who had a considerably higher mortality compared to those with ICU-RRT admission, showing an odds ratio for hospital mortality about 31 times higher. These patients showed the highest benefit from ICU admission in the propensity score adjusted for age and priority level analysis. Thus, the scarcity of beds mainly affected mortality in the group that would benefit the most from ICU-RRT admission^7,8,29^.

In our study, mortality was 69.3%, almost two and a half times higher than the mortality observed in patients who needed RRT (28.4%) in an international multicenter study that evaluated AKI in critically ill patients worldwide^25^. Besides, mortality was exceedingly high in patients who were not admitted to ICU-RRT (82.2%), especially in those with priority level I, of whom 98.0% died. In patients with ICU-RRT admission, hospital mortality was also higher (57.8%) than that observed in the international multicenter study mentioned above^25^. However, the mortality in ICU-RRT admitted patients in our study was lower than that observed in the Brazilian ICUs that participated in another international multicenter study that evaluated AKI associated with critical illness (76.8%)^34^.

Although we did not assess the time between RRT indication and initiation, a significant part of the mortality observed in our study may be due to the delayed RRT initiation after its indication, as patients needed to request treatment in court. Though the proper timing for initiating RRT (early or delayed RRT indication) remains controversial and uncertain^24,35^, in the presence of severe complications such as acute pulmonary edema, severe acidosis, and severe hyperkalemia, RRT should be performed urgently for being the cornerstone of AKI treatment in these patients^35–37^. Moreover, the delayed ICU-RRT admission also postponed the therapeutic and monitoring optimization of the contributing factors of AKI, such as sepsis, the most common etiologic factor of AKI^25,34^. Several studies showed that delayed ICU admission was associated with a worsening in organic dysfunctions and with unfavorable outcomes, such as high mortality, in critical conditions, including septic shock^5,21–23,38,39^. In this respect, patient outcomes in ICU settings can be attributed to the availability of well-trained staff, modern technological resources, and other factors, such as the time elapsed until adequate treatment is established^5,21–23,27,38,39^.

In our study, hospital mortality was lower in patients with renal conditions as the primary reason for hospital admission. In this respect, for having multiple etiologies and risk factors^40^, the pathogenesis of AKI comprises overly complex factors. The effectiveness of ICU admission and RRT undoubtedly depends on the primary reason for hospital admission and on the status of AKI and pre-existing conditions^27,38–41^. Previous studies showed that AKI in sepsis patients is associated with higher mortality rates when compared to patients without sepsis^40,42–44^. Furthermore, the co-existence of nonrenal organ dysfunction, such as acute respiratory failure requiring invasive mechanical ventilation^43^, has been related to worst outcomes in patients with AKI^41,44^. In our study, the most common primary reasons for ICU-RRT admission were cardiovascular and respiratory conditions, commonly related to sepsis, use of invasive mechanical ventilation, non-renal organ dysfunctions, and concomitant occurrence of other organ dysfunctions in addition to AKI. Thus, the lower mortality in patients with renal conditions as the primary reason for hospital admission is in line with previous studies. Besides, history of chronic kidney disease was independently associated with a low mortality in a larger multicenter study that assessed the recognition and management of acute kidney injury^44^.

As observed in our study, although the Brazilian legislation guarantees the right of access to any treatment for every patient, including RRT^30,31^, the scarcity of resources considering the high demand for ICU-RRT admission makes patients wait days for an ICU-RRT bed, while many never get to be admitted^5–8^. Because of this situation, many patients request treatment in court, disregarding the priority established by local regulation centers^30,31^. In our study, although younger age and priority levels I/II were independently associated with a high ICU-RRT admission by court order, more than a half of patients who obtained a favorable court decision had been classified by the Regulation Center that services the Federal District as priority levels III or IV. However, even in patients with ICU-RRT admission, priority level III was independently associated with a high hospital mortality, showing that this group had little benefit from ICU-RRT admission when compared to priority level I and II patients, as shown in the propensity score adjusted for age and priority level analysis. The ICU-RRT admission did not reduce hospital mortality in priority level IV patients. Notwithstanding, priority level I was independently associated with a high mortality only in patients without ICU-RRT admission, not in ICU-RRT admitted patients.

These findings show that, although they may have limitations, the priority criteria for ICU-RRT admission applied by the ICU admission Regulation Center that services the Federal District, which is a governmental authority, were able to identify the highest-risk patients who should benefit from ICU-RRT admission. Furthermore, such triage process puts an end to a highly personal situation of great moral distress, in which ICU and emergency unit physicians had to define which patient should be admitted to ICU-RRT in the shortage of ICU-RRT beds, while the family of patients would demand life support for their relatives^45–48^. Despite these benefits, a significant portion of patients classified as priority levels III and IV who got admitted impaired the admission of patients who would have the most benefit from ICU-RRT treatment. This situation may also lead the patients, families, and ICU staff to deal with a miss triage in the ICU-RRT admission process. Thus, our results reinforce the need for improvement in the ICU triage and rationing, with triage protocols that should be clear and consistent^7,8,31^. To solve this issue, it is also crucial to establish frameworks with triage teams and critical stakeholders, including the participation of judiciary branch representatives, to reduce the number of court litigations that end up in court orders for the ICU-RRT admission of patients with little chance of benefiting from this care, especially in the public health system^39,49^. This effort would result in more patients classified as priority levels I/II having an opportunity to be admitted to ICU-RRT, reducing their mortality. Additionally, the resolution of this scenario of scarce resources for a high demand for ICU-RRT beds also requires the optimization of prevention and treatment strategies by improving the monitoring and treatment of common AKI risk factors and the early AKI diagnosis^24,50^.

Our study has some limitations. First, the data were retrospective. Second, we only evaluated patients classified according to the priority levels established by the ICU admission Regulation Center that services the Federal District. We did not directly assess frailty scores, comorbidity indices, urine outputs, serum levels of creatinine, contributing factors of AKI, need for mechanical ventilation and inotrope/vasopressor support, and commonly used acute illness severity scores, such as the Acute Physiology and Chronic Health Evaluation (APACHE) II scores and the Sequential Organ Failure Assessment (SOFA). Therefore, the difference in mortality among the groups may have been affected by other factors not evaluated in our study.

Regardless of these limitations, our study showed that the variables applied by the Regulation Center that services the Federal District were able to triage patients with a higher chance to benefit from ICU-RRT admission, reinforcing the need for improvement in the ICU-RRT triage and rationing processes. Besides, the shortage of ICU resources, especially RRT support, is a critical problem in several countries, especially in those with a low- and middle-income^6,51,52^. In Brazil, the poorest or less-educated patients who depend exclusively on the public health system are the most affected, as most Brazilian middle- and higher-income residents have a private health insurance. The rich do not use the public health system services, and even if they need initial emergency care in a public service, they can be quickly transferred to an ICU-RRT bed of a private hospital. In fact, most of the judicial litigations in Brazil are filed by public defender's offices that provide legal representation to those less able to pay an attorney^53^. Finally, in the COVID-19 pandemic, these issues were exposed and deepened, with an increased scarcity of ICU resources, such as RRT, and qualified professionals dedicated to the high demand of critically ill patients. The COVID-19 pandemic also brought up innovations: not just the vaccine, but also innovative programs and applications to optimize ICU beds and resources, such as RRT support. Ethical problems also emerged from the choices that have to be made in such a pandemic context. Guidelines for ICU triage and for rationing health resources have been created and legal arguments and laws based on the community's best interest were passed^54^. There has been an increase in the number of ICU beds worldwide, including with RRT support. In fact, from December 2019 to April 2020, there was a 23.59% increase in the number of ICU beds in Brazil^55^. The ICU-RRT bed capacity in the Brazilian public health system needs to be maintained after the COVID-19 pandemic to meet the demand of patients seeking admission. However, the availability of this resource alone will not be enough without an effective ICU-RRT triage system administratively run by a governmental authority. In this respect, future studies may assess artificial intelligence algorithms and artificial neural networks to help those involved in this complex decision. Besides, future studies should also evaluate how the COVID-19 pandemic affected patients critically ill from other conditions and AKI needing RRT, as, although there was an increase in the number of ICU beds and RRT support, many ICUs that used to admit other patients presenting with critical illnesses were restricted only to the admission of patients with COVID-19.

## Conclusion

Patients without ICU-RRT admission showed a much higher hospital mortality than patients with ICU-RRT admission, especially those classified as priority level I. It should be stressed that a large portion of ICU-RRT admitted patients were classified as priority levels III or IV, having little chance to benefit from critical care. These patients had a high mortality even after admission to an ICU-RRT bed, unlike patients classified as priority level I who were admitted to ICU-RRT. Altogether, our findings open new avenues for organizing public policies to improve the prioritization of patients who should be transferred to ICU-RRT, thus optimizing resources, allowing the admission of those who really need it, and, consequently, reducing ICU costs.

## Supplementary Information

Supplementary Tables.

## Data Availability

The authors confirm that the data supporting the findings of this study are available within the article [and/or] its supplementary materials. The data sets used and/or analysed during the current study are available from the corresponding author on reasonable request. The data are not publicly available due to information that could compromise the privacy of research participants.
