# Supplementary material for: Prioritization of ICU beds with renal replacement therapy support by court order and mortality in a Brazilian metropolitan area
Source: Sci Rep. 2022 Mar 3;12:3512. doi: 10.1038/s41598-022-07429-4 (PMC8894379; doi:10.1038/s41598-022-07429-4)
Supplement: Supplementary file 1 — Supplementary Tables. [file 41598_2022_7429_MOESM1_ESM.docx]

**Supplementary Material**

**Prioritization of ICU beds with renal replacement therapy support by court order and mortality in a Brazilian metropolitan area**

*Ana Cristina dos Santos, MSc^1,2^

****corresponding author***

Simone Luzia Fidelis de Oliveira^3,4^

Virgílio Luiz Marques de Macedo^2,5^

Paula Lauane Araujo^2^

Francine Salapata Fraiberg^2^

Nélliton Fernandes Bastos, Nurse^2^

Richard Lucas Alves^6^

Carlos Darwin Gomes da Silveira, MSc^7^

Sérgio Eduardo Soares Fernandes, MSc^7^

Francisco de Assis Rocha Neves, PhD^1^

Fábio Ferreira Amorim, PhD^1,7^

1. Graduation Program in Health Sciences, Universidade de Brasília (UnB), Brasília, DF, Brazil
2. Nursing School, School of Health Sciences, Escola Superior de Ciências da Saúde (ESCS), Brasília, DF, Brazil
3. Regulation Center of Federal District, Secretaria de Saúde do Distrito Federal, Brasília, DF, Brazil
4. Graduation Program in Nursing, Universidade de Brasília (UnB), Brasília, DF, Brazil
5. Multiprofessional Residency Program in Primary Care, Fundação Oswaldo Cruz (FIOCRUZ), Brasília, DF, Brazil
6. Law School, Centro Universitário do Distrito Federal (UDF), Brasília, DF, Brazil
7. Medical School, School of Health Sciences, Escola Superior de Ciências da Saúde (ESCS), Brasília, Brasília, Federal District, Brazil

**Table S1 –** Sample distribution according the public health services in which the patient were treated at the time of the ICU-RRT admission request in court (n = 883)

| Hospital de Base do Distrito Federal | 118 (13.4) |
| --- | --- |
| Hospital Regional de Taguatinga | 93 (10.5) |
| Hospital Regional de Sobradinho | 92 (10.4) |
| Hospital Regional do Gama | 74 (8.4) |
| Hospital Regional de Planaltina | 69 (7.8) |
| Hospital Regional da Asa Norte | 67 (7.6) |
| Hospital Regional de Brazlândia | 58 (6.6) |
| Hospital da Região Leste | 45 (5.1) |
| Hospital Regional de Ceilândia | 37 (4.2) |
| Hospital Regional do Guará | 23 (2.6) |
| Hospital Regional de Samambaia | 21 (2.4) |
| Hospital Regional de Santa Maria | 18 (2.0) |
| Hospital Materno Infantil de Brasília | 0 (0.) |
| Emergency Care Units | 169 (18.0) |

ICU-RRT: Intensive care unit with renal replacement therapy support.

Table S2. Priority level classification according to the criteria of the Regulation Center that services the Federal District.

| Priority level | Criteria |
| --- | --- |
| Priority level I | Critically ill patients who require intensive care should benefit from immediate intensive care/life support interventions and do not have limitations of care. These interventions include invasive and non-invasive mechanical ventilation, vasoactive drugs, and/or other acute interventions.   - Cardiovascular system: complicated acute myocardial infarction with Killip class II or more; cardiogenic shock; complex arrhythmias requiring continuous monitoring and/or acute intervention; acute congestive heart failure with acute respiratory failure and/or hemodynamic support need; hypertensive emergencies with uncontrolled blood pressures after the initial therapy; unstable angina complicated with arrhythmias, hemodynamic instability and/or persistent chest pain; confirmed or presumed aortic dissection; post-cardiac arrest care in patients that do not have limitations of care, cardiac tamponade with hemodynamic instability; or complete heart block or other heart block types with hemodynamic instability that requires a temporary pacemaker. - Respiratory system: clinical or surgical patients with acute respiratory failure requiring non-invasive or invasive mechanical ventilation; severe bronchospasm uncontrolled after the initial therapy; acute pulmonary edema requiring non-invasive or invasive mechanical ventilation; acute pulmonary embolism with hemodynamic instability; or massive hemoptysis. - Nervous system: ischemic stroke in the first four and half hours of symptom onset with the indication for thrombolysis and/or surgical intervention (such as decompressive craniectomy); hemorrhagic stroke with Glasgow Coma Scale bellow nine points, indication for surgical treatment, and/or risk of brain herniation; metabolic coma; acute hypoxic-ischemic brain injury; acute subarachnoid hemorrhage; central nervous system surgery; meningitis with altered mental status or respiratory impairment; intracranial hypertension; central nervous system surgery; severe traumatic brain injury; cerebral vasospasm; refractory seizures; or brain-dead organ donor. - Gastrointestinal system: persistent acute digestive bleeding with hemodynamic instability; fulminant hepatic failure; severe acute pancreatitis; or esophageal perforation. - Renal and urinary system: acute renal failure with urgent renal replacement or associated with severe clinical or surgical acute critical illness (e.g., acute pulmonary edema, hyperkalemia, altered mental status, and circulatory shock). - Oncology and hematology reasons: tumor lysis syndrome. - Endocrine and metabolic reasons: diabetic ketoacidosis with hemodynamic instability; severe electrolyte and acid-base disturbances; thyrotoxic crisis or myxedema coma with hemodynamic instability; or hyperosmolar state with coma and/or hemodynamic instability. - Obstetrics and gynecologic reasons: eclampsia; pre-eclampsia with severe features (formerly severe pre-eclampsia); Hhemolysis, elevated liver enzymes and low platelets (HELLP) syndrome; acute fatty liver of pregnancy; amniotic fluid embolism; antepartum or postpartum hemorrhage; or ovarian hyperstimulation syndrome. - Other reasons: circulatory shock or hemodynamically unstable patients requiring continuous infusion of vasoactive drugs; septic shock; anaphylactic shock; malignant hyperthermia; polytraumatized patients; or disseminated intravascular coagulation. |
| Priority level II | Critically ill patients without hemodynamic instability who require intensive monitoring and care due to the risk of rapid decompensation. They may potentially need immediate intervention and do not have limitations of care.   - Cardiovascular system: uncomplicated acute myocardial infarction with Killip class I; acute arrhythmias requiring hemodynamic stability that requires continuous monitoring; acute congestive heart failure without acute respiratory failure and/or hemodynamic support need; unstable angina without complications, hemodynamic instability, or persistent chest pain; cardiac tamponade with hemodynamic stability; or heart block with hemodynamic stability. - Respiratory system: Chronic obstructive pulmonary disease (COPD) requiring non-invasive mechanical ventilation; respiratory impairment due to non-hypertensive pneumothorax; or acute pulmonary embolism with hemodynamic stability. - Nervous system: ischemic stroke with Glasgow Coma Scale between nine and thirteen points and/or without indication for thrombolysis /or surgical intervention (such as decompressive craniectomy); hemorrhagic stroke with Glasgow Coma Scale between nine and thirteen points without indication for surgical intervention; hemorrhagic stroke without indication for surgical treatment, and/or risk of brain herniation; or meningitis with altered mental status or respiratory impairment. - Gastrointestinal system: acute digestive bleeding with hemodynamic stability that requires continuous monitoring. - Renal and urinary system: acute renal failure without urgent renal replacement.. - Endocrine and metabolic reasons: Uncomplicated hydroelectrolyte and acid-base disorders; or myxedema coma with hemodynamic stability. - Obstetrics and gynecologic reasons: cardiac disease in a pregnant woman; peripartum cardiomyopathy; pre-eclampsia; or thromboembolic disease in pregnancy and the Puerperium. - Another reason: sepsis. |
| Priority level III | Critically ill patients who may require intensive care but have a reduced chance of survival from the underlying disease, the nature of their acute illness, or comorbidities. Intensive treatment may alliviate the critical condition; however, there may be limits on therapeutic efforts, such as intubation or cardiopulmonary resuscitation.   - Oncology and hematology reasons: infection in a patient with metastatic cancer; or neoplasms without therapeutic perspectives of healing. - Nervous system: Ischemic stroke in a patient with previous sequelae and/or with severe comorbidities without acute treatment indication. |
| Priority level IV | Patients with terminal illnesses or who should benefit from palliative care rather than inappropriately aggressive or heroic interventions, and patients who are in good conditions to benefit from ICU admission and are at low risk of needing an intervention that should be performed in an ICU setting.   - Cardiovascular system: mild congestive heart failure; or peripheral vascular surgery. - Respiratory system: chronic obstructive pulmonary disease (COPD) without respiratory failure. - Nervous system: Severe irreversible brain damage; or brain death in a non-organ donor. - Oncology and hematology reasons: metastatic cancer unresponsive to the cancer treatment. - Endocrine and metabolic reasons: diabetic ketoacidosis with hemodynamic stability.   Other reasons: drug overdose without altered mental status; patient awaiting elective surgery with the indication for monitoring in intensive care unit in the immediate postoperative period; irreversible multiorgan dysfunction; permanently unconscious patient. |

**Table S3** – Priority level classification, age, and renal conditions as the primary reason for hospital admission between patients with and without ICU-RRT admission and in patients who requested ICU-RRT admission in court sample before (n = 883) and after matching (n = 736)

| **Variables** | **Before matching** | | | **After matching** | | |
| --- | --- | --- | --- | --- | --- | --- |
|  | **With ICU-RRT admission**  **(n = 476)** | **Without ICU-RRT admission**  **(n = 407)** | ***p-*value** | **With ICU-RRT admission**  **(n = 368)** | **Without ICU-RRT admission**  **(n = 368)** | ***p*-value** |
| Priority level, n (%)  Priority level I  Priority level II  Priority level III  Priority level IV | 154 (32.4)  109 (22.9)  199 (41.8)  14 (2.9) | 101 (24.8)  69 (17.0)  195 (47.9)  42 (10.3) | <0.01 | 101 (47.8)  69 (18.8)  172 (46.7)  26 (7.1) | 85 (23.1)  83 (22.6)  186 (50.5)  14 (3.8) | 0.08 |
| Age, years  mean (SD)  median (IQ 25–75%) | 64.4 (15.2)  70.0 (59.0–79.0) | 67.7 (15.2)  66.0 (55.0–76.0) | <0.01 | 65.8 (15.1)  67.0 (56.0–77.0) | 66.3 (15.7)  68.0 (58.0–77.0) | 0.59 |
| Primary reason for hospital admission, n (%)  Cardiovascular  Respiratory  Renal  Neurological  Digestive  Trauma  Others | 203 (42.6)  132 (27.7)  49 (10.3)  37 (7.8)  29 (6.1)  20 (4.2)  6 (1.3) | 189 (46.4)  96 (23.6)  44 (10.8)  31 (7.6)  34 (8.4)  10 (2.5)  3 (0.7) | 0.38 | 176 (47.80)  88 (23.9)  38 (10.3)  27 (7.3)  28 (7.6)  9 (2.4)  2 (0.5) | 147 (39.3)  114 (31.0)  42 (11.4)  31 (8.4)  18 (4.9)  13 (3.5)  3 (0.8) | 0.15 |

ICU-RRT: Intensive care unit with renal replacement therapy support; SD: standard deviation; IQ 25–75%: interquartile range 25–75%.

**Table S4 –** Age, gender, priority level classification, the primary reason for hospital admission, denial of ICU admission, and hospital mortality in patients who requested ICU-RRT admission in court before matching (n = 883)

| Age, years,  mean (SD)  median (IQ 25–75%) | 65.9 (15.6)  68.0 (57.0–77.0) |
| --- | --- |
| Priority levels, n (%)  Priority level I  Priority level II  Priority level III  Priority level IV | 225 (28.9)  178 (20.2)  394 (44.6)  56 (6.3) |
| Female gender, n (%) | 387 (43.8) |
| Denial of ICU admission, n (%) | 407 (46.1) |
| Primary reason for hospital admission, n (%)  Cardiovascular  Respiratory  Renal  Neurological  Digestive  Trauma  Others | 392 (44.4)  228 (25.8)  93 (10.5)  68 (7.7)  63 (7.1)  30 (3.4)  9 (1.0) |
| Hospital mortality, n (%) | 612 (60.3) |

ICU-RRT: Intensive care unit with renal replacement therapy support; SD: standard deviation; IQ 25–75%: interquartile range 25–75%.

**Table S5 –** Comparision of denial of ICU-RRT admission between priority levels in patients who requested ICU-RRT admission in court (n = 883)

| **Priority level classification** | **Denial of ICU-RRT admission**  **(n = 407)** | **With ICU-RRT admission**  **(n = 476)** | ***p-*value** |
| --- | --- | --- | --- |
| Priority level I, n (%)  Priority level II, n (%)  Priority level III, n (%)  Priority level IV, n (%) | 101 (24.8)  69 (17.0)  195 (47.9)  42 (10.3) | 154 (32.4)  109 (22.9)  199 (41.8)  14 (2.9) | < 0.001 |

ICU-RRT: Intensive care unit with renal replacement therapy support.

**Table S6 –** Comparision of hospital mortality in all patients, ICU-RRT admitted patients and non-ICU-RRT admitted patients according to the pritority level classification in patients who requested ICU-RRT admission in court

| ***Hospital mortality in all patients (n = 883)*** | | | |
| --- | --- | --- | --- |
| **Variables** | **Non-Survivors**  **(n = 612)** | **Survivors**  **(n = 271)** | ***p-*value** |
| Priority level classification, n (%)  Priority level I  Priority level II  Priority level III  Priority level IV | 181 (29.6)  101 (16.5)  310 (50.7)  20 (3.3) | 74 (27.3)  77 (28.4)  84 (31.0)  36 (13.3) | < 0.01 |
| ***Hospital mortality in patients with ICU-RRT admission (n = 476)*** | | | |
| **Variables** | **Non-Survivors**  **(n = 275)** | **Survivors**  **(n = 201)** | ***p-*value** |
| Priority level classification, n (%)  Priority level I  Priority level II  Priority level III  Priority level IV | 82 (29.8)  49 (17.8)  138 (50.2)  6 (2.2) | 72 (35.8)  60 (29.9)  61 (30.3)  8 (4.0) | < 0.01 |
| ***Hospital mortality in patients without ICU-RRT admission (n = 407)*** | | | |
| **Variables** | **Non-Survivors**  **(n = 337)** | **Survivors**  **(n = 70)** | ***p-*value** |
| Priority level classification, n (%)  Priority level I  Priority level II  Priority level III  Priority level IV | 99 (29.4)  52 (15.4)  172 (51.0)  14 (4.2) | 2 (2.9)  17 (24.3)  23 (32.9)  28 (40.0) | < 0.01 |

ICU-RRT: Intensive care unit with renal replacement therapy support; SD: standard deviation; IQ 25–75%: interquartile range 25–75%.
